# Serosurvey and associated risk factors of anti-*Toxocara* spp. antibodies in bovines from slaughterhouses of southeastern Brazil

**DOI:** 10.1186/s13071-021-04755-w

**Published:** 2021-05-11

**Authors:** Paula Andreia Fabris Giudice, Susana Angélica Zevallos Lescano, William Henry Roldan Gonzáles, Rogério Giuffrida, Fernanda Nobre Bandeira, Louise Bach Kmetiuk, Andrea Pires dos Santos, Alexander Welker Biondo, Vamilton Alvares Santarém

**Affiliations:** 1 Graduate College in Animal Sciences, University of Western São Paulo (UNOESTE), Rodovia Raposo Tavares km 572-Bairro Limoeiro, Presidente Prudente, São Paulo, 19050-920 Brazil; 2 Institute of Tropical Medicine of São Paulo, University of São Paulo, São Paulo, 05403-000 Brazil; 3 Department of Veterinary Medicine, Federal University of Paraná, Curitiba, PR 80035-050 Brazil; 4 Department of Comparative Pathobiology, College of Veterinary Medicine, Purdue University, West Lafayette, IN 47907 USA

**Keywords:** Serosurvey, Toxocariasis, Zoonosis

## Abstract

**Background:**

Toxocariasis, caused by a nematode species of the genus *Toxocara*, has been described as one of the most prevalent zoonotic helminthiases worldwide. Human transmission may occur by ingesting *Toxocara* spp. larvae from raw or undercooked meat or organs; however, no comprehensive serosurvey study has been conducted to date investigating the role of cattle as paratenic hosts. The aim of the study reported here was to assess the prevalence of anti-*Toxocara* spp. antibodies and associated risk factors in bovines from two slaughterhouses located in Presidente Prudente, southeastern Brazil.

**Methods:**

Blood samples were collected and tested by indirect enzyme-linked immunosorbent assay (ELISA). Cattle farmers voluntarily responded to an epidemiologic questionnaire.

**Results:**

Overall, 213 of the 553 (38.5%) bovine samples were assessed as seropositive for anti-*Toxocara* spp. antibodies by indirect ELISA. Multivariate analysis revealed that the source of beef cattle and the presence of dogs or cats at the farm were associated with seropositivity. The use of feedlot systems was associated with lower likelihood of seropositivity.

**Conclusions:**

These results indicate a high level of anti-*Toxocara* seropositivity in slaughterhouse cattle, with potentially contaminated meat posing an infection risk to humans. In addition, the presence of dogs and cats where the slaughtered beef cattle were raised was statistically associated with bovine seropositivity, probably due to the overlapping environment at the farm and the lack of pet deworming. The use of feedlot systems was a protective factor likely due to the absence of dog and cat contact, elevated feeding troughs that avoid contact with contaminated soil or grass, and younger age at slaughter of feedlot cattle. In summary, bovines may be used as environmental sentinels of *Toxocara* spp. contamination, and high seropositivity of slaughterhouse cattle may indicate a potential risk of human toxocariasis through the ingestion of raw or undercooked contaminated meat.
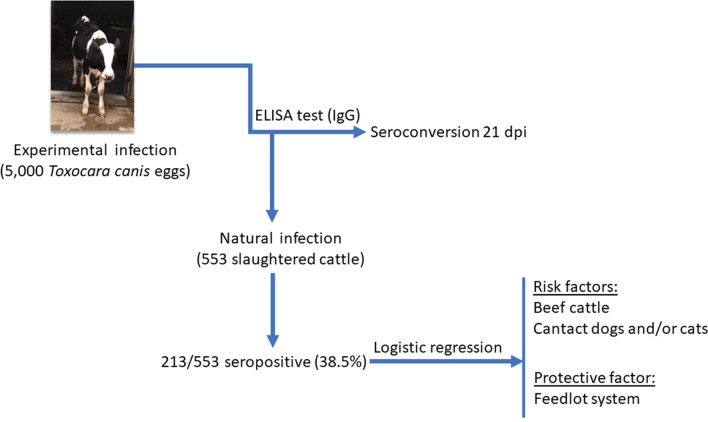

## Background

Toxocariasis is considered to be one of the most prevalent zoonotic helminthiases worldwide, particularly in people of low socioeconomic status [1]. The U.S. Centers for Disease Control and Prevention have considered toxocariasis to be one of the top five neglected parasitic diseases globally with priority for public health actions [2]. Not surprisingly, *Toxocara* seroprevalence has been reported for about one fifth (19.0%) of the global human population, about 1.5 billion people distributed in at least 71 countries [3].

Human toxocariasis is mostly transmitted by ingesting embryonated eggs of *Toxocara* spp. (*T. canis* and *T. cati*) that are shed with the feces of dogs and cats into the environment [1]. The disease's covert form is the most common form of toxocariasis, characterized by chronic nonspecific clinical signs [4, 5] However, migration or larva-induced immune response may lead to the visceral form causing disorders in organs such as the liver and lungs [6], the ocular form, characterized by lesions of the ophthalmologic system [7], or neurotoxocariasis, with invasion of the central nervous system [8].

Although considered an uncommon source, the ingestion of raw or undercooked meat or organs of paratenic hosts, mainly bovines [9–14], is considered a risk factor for human toxocariasis [3]. A case of neuroretinitis with recurrent eosinophilia due to *Toxocara* spp. infection was described in a 36-year Turkish man and linked to his consumption of raw and undercooked meats, including beef [15].

Despite the participation of paratenic hosts in the toxocariasis cycle, their precise role remains a significant gap in our knowledge and understanding of the epidemiology of toxocariasis, specifically its transmission and acquisition [16]. In such a scenario, *Toxocara* (*T*)-seropositivity in paratenic hosts has been essential for a better understanding of transmission among species [16, 17]. Only a few serosurveys have been reported, showing a *T*-seroprevalence of between 13.0 and 51.0% in sheep [18–21], 10.1% in goats [20], 44.6% in horses [22], and from 58.5 to 89.0% in chickens [23–25].

Although human transmission may occur through the ingestion of *Toxocara* spp. larvae in the raw or undercooked meat or organs of paratenic hosts, no comprehensive serosurvey study has been conducted to date in cattle. The aim of the present study was to assess the prevalence of anti-*Toxocara* spp. antibodies and associated risk factors in bovines at two slaughterhouses of Presidente Prudente, southeastern Brazil.

## Methods

The present study was approved (protocol number 3735/2017) by the Research Advisory Committee and by the Ethics Committee of Animal Use of the University of Western São Paulo (UNOESTE).

### Study area

A cross-sectional study was conducted from June 2017 to November 2018 on blood samples collected from bovines slaughtered at two slaughterhouses in the municipality of Presidente Prudente (22°7′16.5540ʺS, 51°23′0.2400ʺW), São Paulo State, southeastern Brazil. Among 5570 Brazilian cities, the city of Presidente Prudente is currently ranked nationally as 126th in terms of population (207,610 inhabitants), 421st in terms of per capita income (US$182.37 per month), and 25th according to the Human Development Index (HDI: 0.806) [26]. In addition, São Paulo State is recorded as having the eighth largest cattle population in Brazil, with approximately 10 million cattle and 3.7 million cattle slaughtered in 2018. Although Presidente Prudente has only 58,638 cattle within the city limits, which is is a highly populated urban area, there are 1.66 million cattle in the surrounding area, of which 1.07 million are beef cattle; 595,000 cattle were slaughtered in 2018 [26]. In the study period, the two slaughterhouses received cattle from 23 farms within the Presidente Prudente region.

### Sampling and questionnaires

The calculated sample size, taking into consideration an estimated prevalence of 15%, error of 3%, and confidence interval (CI) of 95%, was 546 samples [27]. Blood samples were taken from bovines which were at least 24 months old at time of slaughter at one of the two slaughterhouses included in the study. Upon collection, the samples were centrifuged once (1090* g*, 5 min) and the serum subsequently stored at − 20 °C until processing. An enzyme-linked immunosorbent assay (ELISA) was standardized and serum samples tested for the presence of anti-*Toxocara* IgG approximately 2 months after sampling.

Farm owners were asked to complete an epidemiological questionnaire aimed at assessing associated *Toxocara* risk factors. The questionnaire consisted of questions about property, animal rearing system, presence of dogs and cats, and farmr management. More specifically, the questionnaire included questions on: (i) size of the property, categorized as small (up to 96 ha [237 acres]), medium (97–360 ha [890 acres]), and large (> 360 ha [890 acres]); (ii) type of cattle, categorized as beef or dairy; (iii) distance from the main farmhouse to the nearest urban area (≥ 5 km); (iv) cattle rearing system, categorized as extensive for pasture only, semi-extensive for pasture and feeders, and feedlot for full-time confinement; and (v) presence of dogs and/or cats at the farm, and their health management, including the type of food provided (commercial or not, raw meat), hunting habits, last deworming (within 6 months or longer).

### Antibody detection

#### Antigen preparation

The excretory–secretory antigen of *T. canis* larvae was obtained from eggs recovered from sections of the uterus in the anterior portion of female adult nematodes and from eggs naturally shed in infected dog feces. Antigen was extracted following a previously established protocol [18], based on a standard technique [28] with modifications [29]. In short, eggs were kept in 2% formalin for at least 28 days for embryogenesis and washed in physiological solution (0.85% NaCl) by centrifugation (559 *g*, 3 min).

The protein and chitin layers of eggs were removed with 5% sodium hypochlorite, followed by the addition of Eagle media with gentamicin (80 µg/ml). The eggs were ruptured with gentle shaking in an Erlenmeyer flask with glass pearls for 30 min. The solution was kept into sterile tubes at 37 °C, and third larval stage (L3) larvae were recovered using the modified Baermann technique. Fresh Eagle media was added weekly to maintain the L3 larvae. Phenylmethylsulfonyl fluoride (PMSF) at 1 mM was added to the supernatant containing *T. canis* larvae as a protease inhibitor.

The supernatant was then concentrated 50- to 100-fold with commercially available ultrafilters (Amicon, YM10 ultra-centrifugal filter; Merk KGaA, Darmstadt, Germany) and centrifuged (25,155 *g*, 30 min, 4 °C). The supernatant was filtered through a commercial membrane (Millipore membrane filter 0.22-µm pore size; Merk KGaA), and the protein concentration was measured using a the standard method [30]; the filtrate was placed in PMSF and kept at − 20 °C until testing.

Adsorption with antigen extracts from *Ascaris lumbricoides* adult parasites was concomitantly performed to avoid cross-reactivity and ensure reliable results, as previously established [29]. Parasites were washed in distilled water, placed into a porcelain grail, evenly macerated, transferred to a beaker containing 1.5 M sodium hydroxide, and adjusted to a final concentration of 0.15 M. After 2 h at room temperature, the extract was neutralized with 6 N hydrochloric acid, adjusted to pH 7.0, and centrifuged (25,155 *g*, 20 min, 4 °C). The supernatant was filtered through a commercial membrane (Millipore membrane filter 0.22-µm pore size; Merk KGaA), sulfuric acid (at one third of total volume) was mixed with the filtrate and then removed, and the protein concentration was measured using a standard method [30]; the filtrate was kept at − 20 °C until testing.

#### Enzyme-Linked Immunosorbent Assay

Initially, a series of antigens were tested at different concentrations and dilutions, including *T. canis* antigen, commercial peroxidase labeled bovine anti-IgG conjugate (A5295; Sigma-Aldrich, St. Louis, MO, USA), and bovine-positive and -negative controls. The best combination of reagent concentrations were 0.5 µg/ml *T. canis* antigen (100 µg/well), bovine anti-IgG conjugate dilution at 1/10,000, and serum dilution at 1/400.

Polystyrene flatbottom plates were first sensitized with 100 µl of *T. canis* antigen (1.9 µg/ml) diluted in 0.1 M of carbonate buffer pH 9.6, kept at 37 °C for 2 h and then at 4 °C for 18 h. Three washing cycles of 5 min each were performed using commercial phosphate-buffered saline (PBS; 0.01 M pH 7.2 containing 0.05% Tween® [Merk KGaA]). The reaction was blocked with commercial powdered skimmed milk (Molico; Nestlé Co., São Paulo, Brazil) with commercial buffer PBS-T (3% PBS-Tween 20; Thermo Fisher Scientific, Waltham, MA, USA). Plates were kept at 37 °C for 1 h and then washed three times for 5 min each with commercial PBS-T.

Serum adsorption was performed with *A. lumbricoides* extract at 25 μg/ml in 1/200 dilution with PBS-T. Bovine serum samples were then tested in duplicate. Each plate was incubated for 45 min and then washed three times for 5 min each time; bovine anti-IgG conjugate was then added at 1/10,000 dilution in PBS-T, blocked by 100 µl skimmed milk per well, followed by another incubation at 37 °C for 45 min and then three final washes for 5 min each time. Preincubation with antigens of related Ascaridia is a procedure widely adopted to reduce cross-reactive antibodies elicited by exposure to other helminths and, consequently, enhance the specificity of *Toxocara* excretory- secretory antigens based serology [31]. This procedure has been widely applied in ELISA seroprevalence studies involving other paratenic hosts, such as chickens [23–25] and sheep [18, 19, 21]. In our study, each bovine serum sample was treated with an *A. lumbricoides* antigen suspension prior to running the ELISA.

A total of 100 µl of 0.4 mg/ml commercial chromogen solution (Sigma-Fast OPD; Sigma-Aldrich) was added to dry plates along with 0.4 mg/ml hydrogen peroxide urea buffered with 0.05 M phosphate-citrate. The plate was incubated for 30 min in a dark chamber, and the reaction was interrupted with 50 µl of 2.0 M sulfuric acid. The optical density reading was performed at 492 nm using a commercial ELISA reader (Titertek Multiskan MCC/340; Labsystem Diagnostics, Vantaa, Finland).

The reactivity threshold (cut-off) of ELISA was determined using 12 negative samples from bovines born and raised on a farm free of dogs and cats. Optical density average (0.278) with three standard deviations added [(0.278) + 3(0.038)] resulted in a 0.392 cutoff.

#### Positive control

A male, 4-month-old Holstein calf was experimentally infected with 5000 eggs containing *T. canis* larvae and kept in isolation, with at least 2 h of daily sunlight without pasture access. Fecal samples were collected biweekly and tested to ensure the absence of other nematode eggs and protozoan oocysts using the Gordon and Whitlock standard technique [32]. The calf was infected 1 month after weaning, fed twice per day with a balanced, concentrated diet and hay, with water and supplemental salt *ad libidum*, following standard nutritional guidelines.

For calf infection, *T. canis* eggs recovered from sections of the uterus in the anterior portion of female adult nematodes and eggs naturally shed in infected dog feces were used. Eggs were kept in 2% formalin for at least 28 days for embryogenesis and then washed three times in physiological solution (0.85% NaCl) by centrifugation (559 *g*, 3 min) [33]. A total of 5000 eggs were then counted in a Neubauer chamber, followed by dilution in commercial 0.01 M PBS pH 7.2. The solution containing 5000 T*. canis* eggs was administered to the calf* via* an orogastric tube, followed by 100 ml of physiological solution to flush the tube and ensure the full dose was administered. Serum samples were collected prior to experimental infection and then at 7, 14, 21, 28, 40, and 90 days post-infection (dpi) to monitor antibody concentrations.

### Statistical analysis

Positive result percentages by subgroup were estimated with the 95% CI [34]. Outcome data were initially evaluated by univariate analysis (Pearson’s chi-squared test), and variables with a statistical significance of < 0.20 in the univariate model were included in logistical regression analyses to assess risk factors associated with bovine seropositivity. Collinear variables (inflation factor of variance < 4.0) were excluded from the final model. From the regression coefficients for each predictor variable, odds ratio (OR) values were estimated per point together with the 95% CI.

Analyses were conducted using the R statistical program [35] and additional packages [27, 36, 37]. Results with *P* values of < 0.05 were considered to be statistically significant.

## Results

Overall, 213 of the 553 (38.5%, 95% CI 34.5–42.6) bovine samples were assessed as seropositive for anti-*Toxocara* spp. antibodies; these samples were from bovines originating from 22 of the 23 (95.6%) tested farms, with the percentage of seropositive bovines per farm ranging from 5.3 to 90.0% (mean 37.5%) (Table 1). Within the group of seropositive bovines, male bovines showed a higher frequency of anti-*Toxocara* antibodies (163/213; 76.5%) than female bovines (50/213; 23.5%). However, sex was not considered an associated risk factor for antibody presence based on the univariate analysis (OR: 1.2523; 95% CI: 0.8452–1.8556; *P* = 0.26).

**Table 1:** Distribution of the seropositivity for anti-*Toxocara* spp. antibodies in bovines from each farm included in the study

Slaughterhouse	Size of farm	Farm number	Number of samples	Number of positive ELISA results (%)
First^a^	Small	1	19	6 (31.6)
	Small	2	30	5 (16.7)
	Small	3	18	5 (27.8)
	Small	4	8	6 (75.0)
	Medium	5	10	9 (90.0)
Second^b^	Small	6	23	6 (26.1)
	Small	7	18	4 (22.2)
	Small	8	25	2 (8.0)
	Small	9	26	21 (80.8)
	Small	10	15	5 (33.3)
	Medium	11	25	13 (52.0)
	Medium	12	17	5 (29.4)
	Medium	13	25	0 (0.0)
	Medium	14	26	18 (69.2)
	Medium	15	24	7 (29.2)
	Medium	16	33	24 (72.7)
	Large	17	19	1 (5.3)
	Large	18	21	2 (9.5)
	Large	19	24	7 (29.2)
	Large	20	25	14 (56.0)
	Large	21	61	46 (75.4)
	Large	22	31	2 (6.5)
	Large	23	30	5 (16.7)

ELISA, Enzyme-linked immunosorbent assay

^a^Average of 10 bovines slaughtered per day

^b^Average of 300 bovines slaughtered per day

Of the 23 proprieties within the Presidente Prudente region included in the study, nine (39.2%) were considered to be small farming enterprises, seven (30.4%) medium-sized and seven (30.4%) large farms; 20 (87.0%) farms were mainly raising beef cattle; 18 (78.3%) were using semi-extensive cattle systems; and 18 (78.3%) were located > 5 km from urban areas. Almost all farms (21/23; 91.3%) were reported to have dogs, cats, or both on the premises. Regarding the volume of bovines slaughtered at each of the two slaughterhouses, the low-volume slaughterhouse slaughtered an average of ten bovines per day and the high-volume slaughterhouse an average of 300 bovines per day. Bovine samples were collected from slaughtered bovines from five farms at the low-volume slaughterhouse and from slaughtered bovines from 18 farms at the high-volume slaughterhouse.

Univariate analyses (Table 2) showed a positive association of risk factors in medium-sized farms (*P* = 0.0167). Beef cattle systems were 2.37-fold more likely than dairy farm systems to have a positive (*P* = 0.00723) association with anti-*Toxocara* spp. antibodies. A negative (protective) association with anti-*Toxocara* spp. antibodies was found with increasing distance between the farmhouse and urban areas (*P* = 0.0167). Feedlot and semi-confinement systems (*P* < 0.001) were protective factors for toxocariasis. The presence of dogs (*P* < 0.001) or of cats (*P* < 0.001), or both (*P* < 0.001) at the farm was positively associated with positive bovine serology (Table 2).

**Table 2:** Prevalence and associated risk factors for anti-*Toxocara* spp. antibodies (IgG) in bovines (*n* = 553)

Characteristics	ELISA		OR	95% CI	*P* value	Overall *P* value
	Negative,* n* (%)	Positive,* n* (%)				
Size of property						0.167
Small	122 (35.9)	60 (28.2)	Reference			
Medium	84 (24.7)	76 (35.7)	1.84	1.19–2.85	0.00642	
Large	134 (39.4)	77 (36.2)	1.17	0.77–1.78	0.467	
Cattle type						0.00723
Beef	49 (14.4)	14 (6.57)	Reference			
Dairy	291 (85.6)	199 (93.43)	2.37	0.15–2.02	0.00398	
Distance between farmhouse and urban area						0.00106
≤ 5 km	46 (13.5)	53 (24.9)	Reference			
> 5 km	294 (86.5)	160 (75.1)	0.47	0.30–0.73	0.000863	
Cattle raising system						0.00027
Extensive	18 (5.29)	26 (12.2)	Reference			
Semi-extensive	272 (80.0)	174 (81.7)	0.44	0.23–0.83	0.0114	
Feedlot	50 (14.7)	13 (6.1)	0.18	0.08–0.43	0.0000619	
Dogs on farm						0.000732
No	55 (16.2)	13 (6.1)	Reference			
Yes	285 (83.8)	200 (93.9)	2.94	1.61–5.77	0.000296	
Cats on farm						0.000626
No	141 (41.5)	57 (26.8)	Reference			
Yes	199 (58.5)	156 (73.2)	1.93	1.34–2.82	0.000417	
Dogs and cats on farm						0.000000424
No	166 (48.8)	57 (26.8)	Reference			
Yes	174 (51.2)	156 73.2)	2.60	1.80–3.79	0.000000210	

CI, Confidence interval; OR, odds ratio

Multivariate analysis (Table 3) showed a positive association between risk factors for *Toxocara* spp. ELISA seropositivity in beef cattle (*P* < 0.001) and presence of dogs (*P* = 0.006) and cats (*P* < 0.001) on the farm, and a negative (protective) association with feedlot systems (*P* < 0.001). The presence of dogs and cats at the farm showed collinearity with the presence of cats alone and was excluded from multivariate analysis.

**Table 3:** Multivariate analysis of risk factors associated with anti-*Toxocara* spp. antibodies (IgG) in bovines

Characteristics	Estimate	Standard error	*z*	Pr( >|z|)	OR (95% CI)
Intercept	− 1.3048	0.5869	− 2.223	0.0662	NA
Size of property	− 0.1252	0.1353	− 0.925	0.355054	0.8824 (0.6766–1.1514)
Beef cattle	0.9924	0.3685	2.693	0.00707	2.6978 (1.3315–5.6913)
Distance between farmhouse and urban area	− 0.3648	0.2566	− 1.422	0.15512	0.6944 (0.4192–1.1483)
Feedlot	− 1.0013	0.2388	− 4.194	0.0000275	0.3674 (0.2265–0.5794)
Presence of dogs	0.9198	0.3381	2.721	0.00652	2.5088 (1.3271–5.0431)
Presence of cats	0.8522	0.2236	3.811	0.000139	2.3448 (1.5235–3.6675)

NA, Not applicable; * z*, statistic for Wald tests that measures the ratio between the coefficient and its standard error

In dogs, univariate analysis showed an association of bovine *T*-seropositivity with dogs fed with non-commercial food (OR: 2.09, 95% CI 1.443–3.028; *P* < 0.001), raw meat intake (OR: 4.88, 95% IC 3.14–7.72; *P* < 0.001), and hunting habits (OR : 2.10, 95% CI 1.44–3.08; *P* < 0.001). Analysis of the completed questionnaire showed that ten of the 23 farms (43.5%) reported dog deworming within the past 6 months, with no difference between those farms reporting deworming at > 6 months (OR: 0.97, 95% CI 0.6739–1.389; *P* = 0.931). Due to a lack of consistency of questionnaire answers, no analysis was performed on the health habits of cats.

There was also no statistical difference in seropositivity between bovines slaughtered at each of the slaughterhouses (OR: 0.9021, 95% CI 0.559–1.459; *P* = 0.7173), i.e., between bovines sampled in the low-volume (31/85; 31%) and high-volume slaughterhouse (182/468; 33%).

**Fig. 1:**
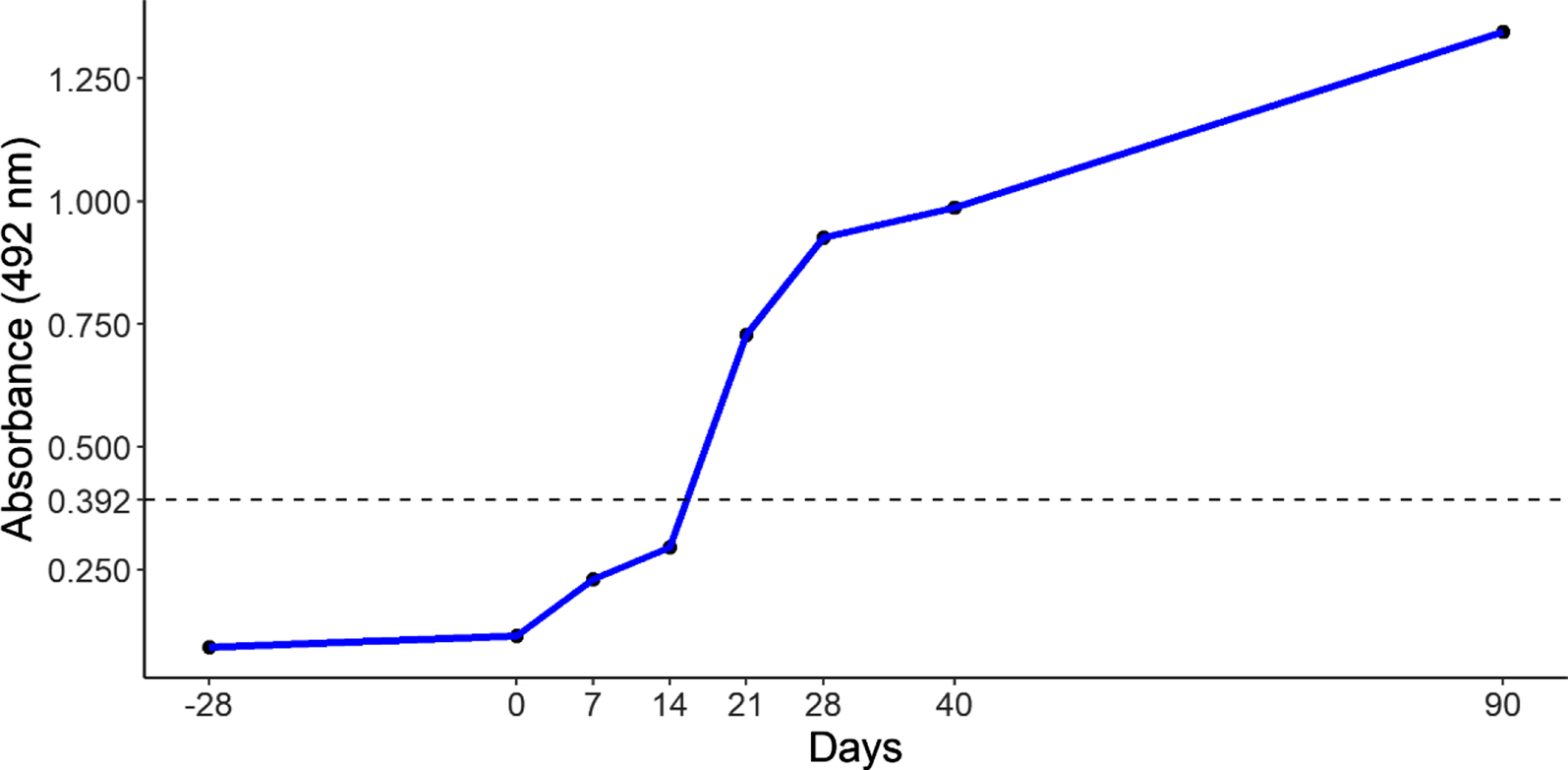
Kinetics of anti-*Toxocara canis* antibodies (IgG) in an experimentally infected bovine. Absorbance was read at a wavelength of 492 nm with a cut-off value of 0.392 (dotted horizontal line). Serological tests were conducted from 28 days prior to experimental infection until 90 days post-infection

Seroconversion of an experimentally infected calf was observed at 21 dpi, with increasing optical density from 7 to 90 dpi (end of monitoring period) (Fig. 1).

## Discussion

To the authors’ knowledge, this is the first serosurvey of *Toxocara* spp. in cattle. The study reports *T-*seroprevalence in bovines, showing relatively high prevalence, with an overall seropositivity of about 40%. We consider the estimated prevalence to be relatively high, suggesting that beef cattle are frequently exposed to *Toxocara* spp*.* and are able to participate in transmission cycles associated with the consumption of undercooked meat. Analysis of our data revealed a wide variation in prevalence among the bovine farms studied, ranging from one of 19 bovines (5.26%) to nine of ten bovines (90.0%); This variation is in agreement with the results of a previous study on sheep farms in southern Brazil that also showed variations in farms for *T*-seropositivity, ranging from two of 17 sheep (11.7%) to ten of 15 sheep (66.6%) [21].

High *T-*seroprevalence (88 positive samples in a total of 188; 44.6%) was also found in a study of horses of Mexico [22], with stall-raised sporthorses (no pasture access) being 5.4-fold less likely to be positive than horses raised on pasture for human consumption. A similar outcome was observed in the present study, as feedlot bovines were 5.6-fold less likely to be seropositive that those raised in extensive systems with continuous pasture contact. In addition, bovines may enter the feedlot system at an earlier age, around 12–24 months [38], with a lower lifetime until slaughter and therefore less time for exposure to *Toxocara* spp.

Another factor for exposure to *Toxocara* spp. eggs was the mode of pasture foraging. In one study, sheep, which mostly consume creeping grasses, were reported to have a 7.7-fold higher odds of *Toxocara* spp. infection than goats, which mainly eat from bushes [20]. Similar to most of Brazil, the pasture in the study region was mostly covered by *Brachiaria* spp., a creeping grass extensively cultivated as tropical forage for beef cattle.

The presence of dogs and cats at the farm was also an associated risk factor for *T*-seropositivity in bovines. Similar outcomes have been observed for other dog and cat parasites transmitted to herbivores through environmental ingestion of eggs or protozoa oocysts, such as *Neospora caninum* and *Toxoplasma gondii* [39, 40]. Not surprisingly, bovines raised in farms along with dogs were 2.84-fold more susceptible to *N. caninum* infection [41]. In this study, at only one farm, without dogs and cats, were all of the bovine samples seronegative, highlighting the role of companion animals in contaminating the environment with *Toxocara* spp. eggs and leading to bovine infection.

In the present study, farms with dogs that consumed raw meat and hunted were more likely to have cattle that were seropositive for *Toxocara* spp. Ingestion of raw meat and organs from paratenic hosts by dogs and cats may complete and maintain the nematode cycle [17]. Since dogs fed with non-commercial food may imply homemade food or leftovers, including raw meat intake, such dogs may have greater propensity for reinfection by *T. canis* eggs from paratenic hosts or at their living place [17, 42].

No association was found between dog deworming and bovine seropositivity for *Toxocara* spp.; however, most farmers completing the questionnaire were uncertain about the date of the last anti-helminthic treatment, the identify of the drug, and the dose used. Regardless, environmental contamination and exposure of definitive and paratenic hosts with *Toxocara* spp. have been confirmed, emphasizing the importance of farmer conscientization of their role on animal and human health [16].

Although the analytical results in this study indicate that beef cattle were more likely to be infected by *Toxocara* spp. than dairy cattle, a previous study reported that the opposite was true for infection by *Neospora caninum*, with dairy cattle found to be 1.6-fold more likely to be infected than beef cattle [41]. This difference may be explained by the higher susceptibility of dairy cattle to *N. caninum* infection [41], which has not reported for *Toxocara* spp. to date. The authors of that study hypothesized that environmental contact with contaminated soil may be the most critical factor and, therefore, that confinement of dairy cattle to feedlots may be protective as well.

Our results indicate a high level of anti-*Toxocara* seropositivity in bovines slaughtered and destined for human consumption in two official slaughterhouses subjected to federal and/or city inspections. As human socioeconomic and hygiene conditions of subsistence production may contribute to the transmission cycle of meat-borne diseases [43, 44], *Toxocara* spp. prevalence may be even higher in bovines slaughtered at unofficial slaughterhouses or in backyard slaughter. A recent meta-analysis study showed that consuming raw or undercooked meat, mostly beef [3], is a risk factor for human toxocariasis [3]. Moreover, feeding pets with raw meat may perpetuate the life-cycle of these nematodes [19].

A limitation of this study is that *T. canis* may not be clearly distinguishable by traditional serological diagnostic methods from *Toxocara vitulorum* [45] for which water buffalo (*Bubalus bubalis*) of tropical and subtropical geographical areas are the main reported hosts [46]. However, *T. vitolurum* reports in Brazil have been restricted to buffalo calves, with no infection described to date in either adult buffalo or cattle [47, 48]. Moreover, no farm in the present study had buffalo or was located close to a buffalo or mixed buffalo–cattle farm, and in 2019, only 195 (0.33%) water buffalo were reported within city limits, compared to 58,638 cattle [26]. Nevertheless, future serological studies of *T. canis* in Brazilian areas of overlapping cattle and water buffalo farms should also consider *T. vitulorum* cross-reactivity.

Although the aim of the present study was not to detect viable *Toxocara* spp. larvae in bovine meat, the overall seropositivity may strongly indicate their presence in fresh beef. Thus, further studies should be conducted to fully establish the role of bovines on *Toxocara* spp. epidemiology, the concurrent exposure and associated risk factors of the local human population, and the serological status of other Brazilian regions.

## Conclusions

In conclusion, our results indicate a high level of anti-*Toxocara* seropositivity in slaughterhouse cattle, with potentially contaminated meat posing an infection risk to humans. The presence of dogs and cats, beef cattle, and extensive systems were identified as risk factors, while feedlot systems were a protective factor. Bovines may be used as sentinels of environmental contamination with *Toxocara* spp., and the seropositivity of slaughterhouse cattle may indicate a potential risk of human toxocariasis through the ingestion of raw or undercooked contaminated meat.

## Data Availability

The datasets used and/or analyzed during the current study are available from the corresponding author on reasonable request.

## References

[1] Ma G, Holland CV, Wang T, Hofmann A, Fan C-K, Maizels RM, et al. Human toxocariasis. Lancet Infect Dis. 2018;18(1):e14-24. 10.1016/S1473-3099(17)30331-6 28781085

[2] U.S. Centers for Disease Control and Prevention (CDC). Toxocariasis. https://www.cdc.gov/parasites/toxocariasis/. Accessed 5 Jan 2021

[3] Rostami A, Riahi SM, Holland CV, Taghipour A, Khalili-Fomeshi M, Fakhri Y, et al. Seroprevalence estimates for toxocariasis in people worldwide: a systematic review and meta-analysis. PLoS Negl Trop Dis. 2019;13(12):e0007809. 10.1371/journal.pntd.0007809. 10.1371/journal.pntd.0007809 PMC6922318 31856156

[4] Gavignet B, Piarroux R, Aubin F, Millon L, Humbert P (2008). Cutaneous manifestations of human toxocariasis. J Am Acad Dermatol.

[5] Poulsen CS, Skov S, Yoshida A, Skallerup P, Maruyama H, Thamsborg SM, et al. Differential serodiagnostics of *Toxocara canis* and* Toxocara cati*—is it possible? Parasite Immunol. 2015;37(4):204–7. 10.1111/pim.12181 25711956

[6] Arslan F, Baysal NB, Aslan A, Simsek BC, Vahaboglu H (2019). Toxocara related peritonitis: a case report and review of literature. Parasitol Int.

[7] Zibaei M, Alemi M, Cardillo NM, Derafshi H, Miahipour A, Bahadory S, et al. Human toxocariasis seroprevalence among patients with uveitis in Alborz Province. Iran Ann Agric Environ Med. 2019;26(1):154–8. 10.26444/aaem/102293. 10.26444/aaem/102293 30922047

[8] Nicoletti A (2020). Neurotoxocariasis. Adv Parasitol.

[9] Mitsuhashi Y, Naitou K, Yamauchi S, Naruse H, Matsuoka Y, Nakamura-Uchiyama F, et al. A case of the myelitis due to* Toxocara canis* infection complicated with cervical spondylosis. No Shinkei Geka. 2006;34(11):1149–54. 17087270

[10] Mitamura M, Fukuoka M, Haruta Y, Koarada S, Tada Y, Nagasawa K (2007). A case of visceral larva migrans due to Toxocara canis showing varied manifestations. Kansenshogaku Zasshi [J Japanese Assoc Infect Dis].

[11] Yoshikawa M, Nishiofuku M, Moriya K, Ouji Y, Ishizaka S, Kasahara K (2008). A familial case of visceral toxocariasis due to consumption of raw bovine liver. Parasitol Int.

[12] Choi D, Lim JH, Choi D-C, Lee KS, Paik SW, Kim S-H (2012). Transmission of Toxocara canis via ingestion of raw cow liver: a cross-sectional study in healthy adults. Korean J Parasitol.

[13] Kwon HH (2015). Toxocariasis: a rare cause of multiple cerebral infarction. Infect Chemother..

[14] Deshayes S, Bonhomme J, de La Blanchardière A (2016). Neurotoxocariasis: a systematic literature review. Infection.

[15] Karaca I, Mentes J, Nalçacı S (2018). Toxocara neuroretinitis associated with raw meat consumption. Turkish J Ophthalmol.

[16] Holland CV (2017). Knowledge gaps in the epidemiology of Toxocara: the enigma remains. Parasitology.

[17] Strube C, Heuer L, Janecek E (2013). *Toxocara* spp. infections in paratenic hosts. Vet Parasitol..

[18] Lloyd S (2006). Seroprevalence of *Toxocara canis* in sheep in Wales. Vet Parasitol.

[19] Santarém VA, Chesine PAF, Lamers BEL, Rubinsky-Elefant G, Giuffrida R (2011). Anti-*Toxocara* spp. antibodies in sheep from southeastern Brazil. Vet Parasitol..

[20] Kantzoura V, Diakou A, Kouam MK, Feidas H, Theodoropoulou H, Theodoropoulos G (2013). Seroprevalence and risk factors associated with zoonotic parasitic infections in small ruminants in the Greek temperate environment. Parasitol Int.

[21] Rassier GL, Borsuk S, Pappen F, Scaini CJ, Gallina T, Villela MM (2013). *Toxocara* spp. seroprevalence in sheep from southern Brazil. Parasitol Res..

[22] Heredia R, Romero C, Mendoza GD, Ponce M, Carpio JC (2018). Identifying anti-*Toxocara* IgG antibodies in horses of Mexico. Arq Bras Med Vet Zootec.

[23] Campos-da-Silva DR, da Paz JS, Fortunato VR, Beltrame MAV, Valli LCP, Pereira FEL (2015). Natural infection of free-range chickens with the ascarid nematode *Toxocara* sp. Parasitol Res.

[24] von Söhsten AL, da Silva AV, Rubinsky-Elefant G, Guerra LMS de MEM. Anti-*Toxocara* spp IgY antibodies in poultry sold in street markets from Feira de Santana, Bahia, Northeastern Brazil. Vet Parasitol Reg Stud Rep. 2017;8:86–9. 10.1016/j.vprsr.2017.02.006 10.1016/j.vprsr.2017.02.006 31014645

[25] de Oliveira AC, Rubinsky-Elefant G, Merigueti YFFB, Batista A da S, Santarém VA. Frequency of anti-*Toxocara* antibodies in broiler chickens in southern Brazil. Rev Bras Parasitol Vet. 2018;27(2):141–5. 10.1590/s1984-296120180025 29846447

[26] Brasil/ São Paulo/ Presidente Prudente. Instituto Brasileiro de Geografia e Estatística. 2019. https://cidades.ibge.gov.br/brasil/sp/presidente-prudente/pesquisa/18/16459. Accessed 5 May 2021

[27] Hebbali A. Tools for developing binary logistic regression models [R package blorr version 0.3.0]. 2020. https://cran.r-project.org/package=blorr. Accessed 5 Jan 2021

[28] Savigny DH (1975). In vitro maintenance of Toxocara canis larvae and a simple method for the production of Toxocara ES antigen for use in serodiagnostic tests for visceral larva migrans. J Parasitol.

[29] Elefant GR, Shimizu SH, Sanchez MCA, Jacob CMA, Ferreira AW (2006). A serological follow-up of toxocariasis patients after chemotherapy based on the detection of IgG, IgA, and IgE antibodies by enzyme-linked immunosorbent assay. J Clin Lab Anal.

[30] Lowry OH, Rosebrough NJ, Farr AL, Randall RJ (1951). Protein measurement with the Folin phenol reagent. J Biol Chem.

[31] Rubinsky-Elefant G, Hirata CE, Yamamoto JH, Ferreira MU (2010). Human toxocariasis: diagnosis, worldwide seroprevalences and clinical expression of the systemic and ocular forms. Ann Trop Med Parasitol.

[32] Ueno H, Gonçalves PC (1998). Manual para diagnóstico de helmintoses de ruminantes.

[33] Pecinali NR, Gomes RN, Amendoeira FC, Bastos ACMP, Martins MJQA, Pegado CS (2005). Influence of murine Toxocara canis infection on plasma and bronchoalveolar lavage fluid eosinophil numbers and its correlation with cytokine levels. Vet Parasitol.

[34] Agresti A, Coull BA (1998). Approximate is better than “Exact” for interval estimation of binomial proportions. Am Stat.

[35] The R project for statistical computing. https://www.r-project.org/. Accessed 5 Jan 2021.

[36] Manning C. Logistic regression (with R). 2007. http://nlp.stanford.edu/manning/courses/ling289/logistic.pdf.. Accessed 20 Mar 2021.

[37] Subirana I, Sanz H, Vila J (2014). Building Bivariate tables: the compareGroups package for R. J Stat Softw.

[38] Senturklu S, Landblom D, Maddock R, Petry T, Wachenheim C, Paisley S (2018). Effect of yearling steer sequence grazing of perennial and annual forages in an integrated crop and livestock system on grazing performance, delayed feedlot entry, finishing performance, carcass measurements, and systems economics. J Anim Sci.

[39] Bruhn FRP, Daher DO, Lopes E, Barbieri JM, da Rocha CMBM, Guimarães AM (2013). Factors associated with seroprevalence of *Neospora caninum* in dairy cattle in southeastern Brazil. Trop Anim Health Prod.

[40] Appelt MA, da Silva AS, Cazarotto CJ, Machado G, Rodrigues RS, Norbury LJ (2019). Cholinesterase as an inflammatory marker of subclinical infection of dairy cows infected by Neospora caninum and risk factors for disease. Comp Immunol Microbiol Infect Dis.

[41] Ribeiro CM, Soares IR, Mendes RG, de Santis Bastos PA, Katagiri S, Zavilenski RB (2019). Meta-analysis of the prevalence and risk factors associated with bovine neosporosis. Trop Anim Health Prod.

[42] Nijsse R, Mughini-Gras L, Wagenaar JA, Franssen F, Ploeger HW (2015). Environmental contamination with Toxocara eggs: a quantitative approach to estimate the relative contributions of dogs, cats and foxes, and to assess the efficacy of advised interventions in dogs. Parasit Vectors.

[43] Macpherson CNL (2013). The epidemiology and public health importance of toxocariasis: a zoonosis of global importance. Int J Parasitol.

[44] Pozio E (2020). How globalization and climate change could affect foodborne parasites. Exp Parasitol..

[45] Mahdy OA, Mousa WM, Abdel-Maogood SZ, Nader SM, Abdel-Radi S (2020). Molecular characterization and phylogenetic analysis of toxocara species in dogs, cattle and buffalo in Egypt. Helminthologia.

[46] Roberts JA (1989). The extraparasitic life cycle of Toxocara vitulorum in the village environment of Sri Lanka. Vet Res Commun.

[47] Silva D, Santana A, Pizauro LJL, Bernardes P, Clemente V, Silveira C (2015). Toxocara vitulorum in newborn buffalo calves. Investigação.

[48] Ribeiro MG, Langoni H, Jerez JA, Leite D da S, Ferreira F, Gennari SM. Identification of enteropathogens from buffalo calves with and without diarrhoea in the Ribeira Valley, State of São Paulo, Brazil. Braz J Vet Res Anim Sci. 2000;37(2).

